# Paper-based wearable electronics

**DOI:** 10.1016/j.isci.2021.102736

**Published:** 2021-06-17

**Authors:** Yadong Xu, Qihui Fei, Margaret Page, Ganggang Zhao, Yun Ling, Samuel B. Stoll, Zheng Yan

**Affiliations:** 1 Department of Biomedical, Biological & Chemical Engineering, University of Missouri, Columbia, MO 65211, USA; 2 Department of Mechanical & Aerospace Engineering, University of Missouri, Columbia, MO 65211, USA

**Keywords:** Biodevices, Bioelectronics, Electronic materials

## Abstract

Skin-interfaced wearable electronics can find a broad spectrum of applications in healthcare, human-machine interface, robotics, and others. The state-of-the-art wearable electronics usually suffer from costly and complex fabrication procedures and nonbiodegradable polymer substrates. Paper, comprising entangled micro- or nano-scale cellulose fibers, is compatible with scalable fabrication techniques and emerges as a sustainable, inexpensive, disposable, and biocompatible substrate for wearable electronics. Given various attractive properties (e.g., breathability, flexibility, biocompatibility, and biodegradability) and rich tunability of surface chemistry and porous structures, paper offers many exciting opportunities for wearable electronics. In this review, we first introduce the intriguing properties of paper-based wearable electronics and strategies for cellulose modifications to satisfy specific demands. We then overview the applications of paper-based devices in biosensing, energy storage and generation, optoelectronics, soft actuators, and several others. Finally, we discuss some challenges that need to be addressed before practical uses and wide implementation of paper-based wearable electronics.

## Graphical abstract

**Figure:**
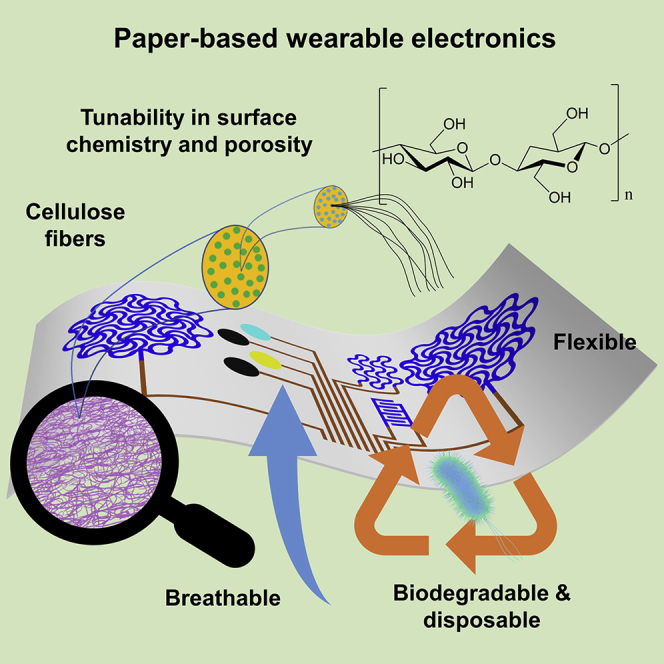

## Introduction

Emerging wearable electronics have achieved significant advancements in a wide spectrum of applications, spanning from healthcare and human-machine interface to soft robots and virtual and augmented reality (Bariya et al., 2018; Kim et al., 2019; Ray et al., 2019; Someya and Amagai, 2019; Someya et al., 2016; Yang and Gao, 2019). Existing wearable electronics usually rely on clean-room-based fabrication techniques and/or nonbiodegradable polymer supporting substrates (e.g., polyimide [PI], polyethylene terephthalate [PET], and silicone elastomers) (Liu et al., 2017; Sun et al., 2018; Xu et al., 2020a; Yu et al., 2019). Its commercial translation is therefore limited as a result of the high manufacturing cost. Moreover, next-generation wearable electronics should be skin friendly (e.g., breathable), disposable, and one-time use to minimize the risks of inflammation and infections. Considering the wide distribution and implementation of wearable electronics in the future, the accumulation of disposed electronic wastes will require substantial demand for the landfill space and cause unfavorable environmental issues. Such requirements contradict the current materials selections of wearable electronics as they are generally nonbreathable, nonbiodegradable, and expensive. Therefore, researchers are motivated to seek sustainable alternatives with desired features that can address the aforementioned handicaps.

**Figure 1:**
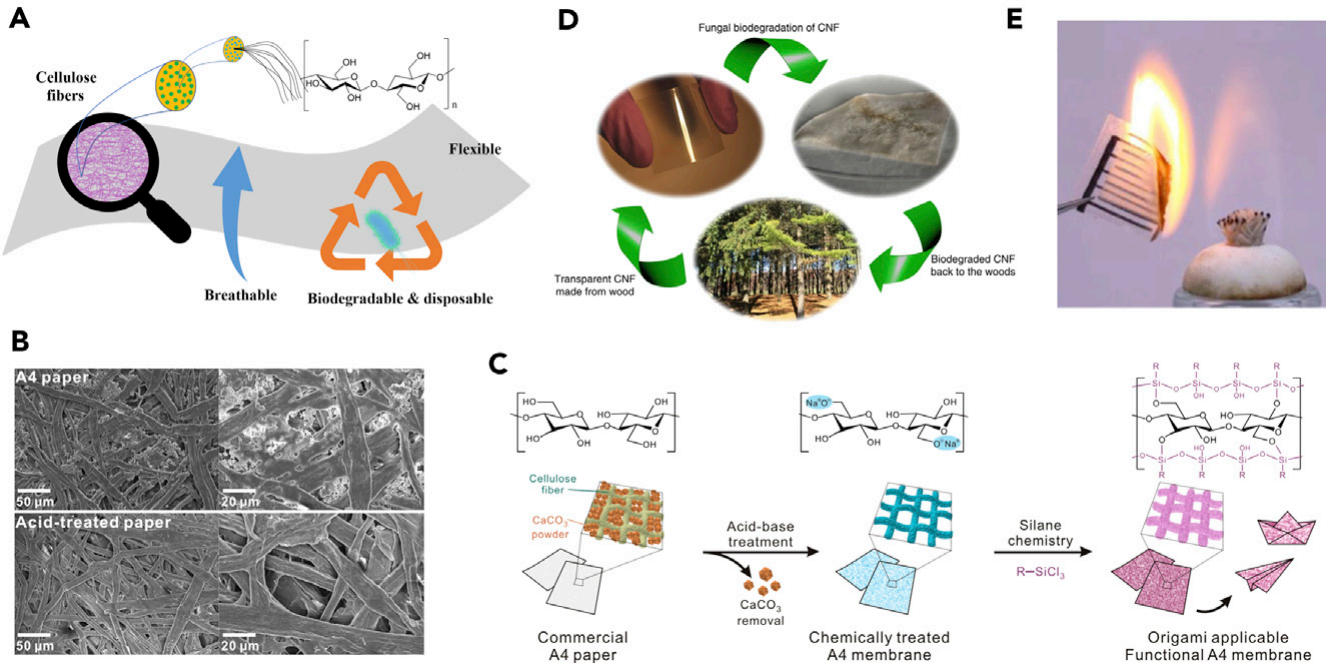
An overview of desirable merits of cellulose paper (A) Schematic illustration of cellulose paper with high flexibility, breathability, biodegradability, and disposability. (B) Scanning electron microscope (SEM) images of pristine and acid-treated commercial paper. Adapted with permission from Ahn et al. (2020). Copyright 2020, American Chemical Society. (C) Acid-base treatment and surface modification of cellulose paper with trichlorooctylsilane. Adapted with permission from Ahn et al. (2020). Copyright 2020, American Chemical Society. (D) A typical life cycle of the biodegradable, eco-friendly, and sustainable cellulose paper. Adapted with permission from Jung et al. (2015). Copyright 2015, Nature Publishing Group. (E) Incineration of a paper-based electronic device. Adapted with permission from Gao et al. (2019a). Copyright 2019, American Chemical Society.

Paper is mainly composed of cellulose microfibers with diameters of tens of microns and lengths up to 5 mm, which can be further extracted into nanofibrils with substantially decreased diameters consisting of ordered linear cellulose molecular chains (Figure 1A) (Mahadeva et al., 2015; Tobjörk and Österbacka, 2011). The abundance of raw materials from wood, together with scalable roll-to-roll (R2R) manufacturing process, enables the extensive use of low-cost cellulose paper (0.1 cent dm^−2^) in our daily life (Tobjörk and Österbacka, 2011). Examples include packaging, display, and information storage. Owing to the hierarchically entangled cellulose fiber structures, paper features many intriguing properties in terms of rich surface chemistry (i.e., hydrophilic hydroxyl groups) and pore size tunability. Moreover, the superior tailorability of paper granted by kirigami and origami designs brings more possibilities for the development of paper-based three dimensional devices (Ding et al., 2016; Lin et al., 2017; Xu et al., 2020b). In addition, paper's intrinsically sustainable, breathable, flexible, biocompatible and biodegradable nature broadens its promising and versatile applications in wearable electronics (Figure 1A). Leveraging scalable manufacturing approaches (e.g., inkjet printing, screen printing) or highly customizable and easily accessible writing process (e.g., pencil or pen writing), myriads of applications have been explored for paper-based wearable electronics, including displaying (Asadpoordarvish et al., 2015; Ha et al., 2018; Jeong et al., 2019), sensing (Mahadeva et al., 2015; Tai et al., 2020), optoelectronics (Ha et al., 2018), energy harvesting and storage (Yao et al., 2017; Zhang et al., 2015), and many others (Hamedi et al., 2016; Liao et al., 2020).

In this review, we discuss the potential, progress, and challenges of cellulose paper-based wearable electronics. Specifically, we first introduce the critical mechanical and chemical features of cellulose papers, which can meet the requirements of human- and eco-friendly wearable electronics. Next, we overview the recent research of paper-based electronic devices, including biosensing, energy harvesting and storage, soft actuators, optoelectronics, and several others. Finally, we discuss the opportunities and future challenges. We believe that paper-based wearable electronics can find a breath of potential applications in low-resource environments and home-based, personalized healthcare as a sustainable platform.

## Property demand

Wearable electronics require mechanically flexible and stretchable yet robust supporting substrates. At the molecular level, the inter- and intramolecular hydrogen bonds among densely packed hydroxyl groups, together with Van del Waals interactions, in cellulose paper generate strong interfacial interactions (Li et al., 2021). Moreover, randomly distributed fibrous networks provide physical entanglement that forms porous structures (Figure 1B), which further enhance the flexibility (Ahn et al., 2020). These features offer lightweight, flexible, yet mechanically tough properties. Commercial copy paper is typically filled with mineral fillers such as calcium carbonate (CaCO_3_), chalk, and clays to improve light scattering, ink absorbency, and surface smoothness (Tobjörk and Österbacka, 2011). Removing these fillers can tune the porosity, pore size distribution, and mechanical strength. For example, acid and/or base treatments of a commercial A4 paper increase the pore size and porosity from 0.31 μm and 50.3% to 12.2 μm and 82.9% (Figures 1B and 1C) (Ahn et al., 2020). As a result, the mechanical toughness along with fracture strain decreased somewhat, whereas the tensile strength increased from ∼20 to ∼30 MPa. In addition, the pristine porous fibrous network provides excellent breathability (>6,000 g m^−2^ day^−1^) (Xu et al., 2020b) that far surpasses that of human skin (204 g m^−2^ day^−1^) (Chao et al., 2018), which is critical considering long-term and continuous skin-interfaced applications. Also, high stretchability can be achieved in paper with judiciously engineered kirigami designs.

The abundant hydroxyl groups of cellulose fibers are advantageous for microfluidics where liquid transportation is driven by capillary force. The rich surface chemistry has also been explored when integrating paper with conductive materials (e.g., metallic nanomaterials, carbonaceous nanomaterials) (Zhang et al., 2018). However, the intrinsic hygroscopic nature of cellulose paper inevitably compromises the stability and robustness because of unfavorable mechanical degradation and irreversible deformations in wet environments (Tobjörk and Österbacka, 2011). This handicap can be resolved by surface chemistry engineering. For example, silanization of paper results in substantially increased hydrophobicity (Figure 1C) (Ahn et al., 2020). Particularly, an omniphobic “R^F^” paper was fabricated by vapor-phase silanization of paper with fluoroalkyl trichlorosilanes, while preserving its intrinsic porous structure (Glavan et al., 2014). This strategy enables a hydrophobic and oleophobic paper that repels both aqueous and organic solutions such as blood, significantly improving the stability and robustness in biological environments. Inkjet printing of conductive inks on the “R^F^” paper also exhibits higher lateral printing resolution down to ∼28 μm (Lessing et al., 2014). In addition, other device fabrication methods, which generally involve deposition of hydrophobic reagents on paper, are also demonstrated, including analog printing, wax printing, flexography printing, and screen printing (Mahadeva et al., 2015).

Given the potential of green and environmentally friendly wearable electronics, cellulose paper is playing increasingly critical role as a sustainable supporting substrate. While most polymers take hundreds of years to decompose in natural environments, cellulose paper can be decomposed within several weeks by a variety of microorganisms (fungi, bacteria, and yeasts) that exist naturally in soil (Coughlan, 1991). Figure 1D presents a typical life cycle of cellulose nanofibrils that are originally extracted from wood, subsequently degraded by fungi, and recycled back for forestry fertilizer (Jung et al., 2015). Specifically, two types of fungi, brown rot fungus *Postia placenta* and white rot fungus *Phanerochaete chrysosporium*, have demonstrated the capability of cellulose biodegradation. The disposability of paper-based electronics is also exemplified by a simple incineration process (Figure 1E) (Gao et al., 2019a). Moreover, cellulose is considered as a biocompatible material and has been used in numerous bioengineering-related fields. In addition to skin-interfaced electronic devices that are covered in this review, other application areas of cellulose materials include tissue engineering, drug delivery, medical microfluidic diagnostics, and wound healing (Cate et al., 2015; Czaja et al., 2006; Gong and Sinton, 2017; Hickey and Pelling, 2019; Sun et al., 2019). Owing to the aforementioned properties, cellulose paper emerges as an appealing platform for the fabrication of wearable electronics.

## Applications of paper-based wearable electronics

### Biosensing

Wearable electronics can provide continuous, long-term monitoring of dynamic changes of physiological signals and hold great promise in fitness tracking, medical diagnostics, and human-machine interface (Bariya et al., 2018; Kim et al., 2019; Ray et al., 2019; Someya and Amagai, 2019; Someya et al., 2016; Yang and Gao, 2019). Owing to the intrinsic nonconductive nature (10^11^–10^15^ Ω sq^−1^ at a relative humidity of 20%–40%) of cellulose paper (Tobjörk and Österbacka, 2011), conductive and semiconductive materials are required to make paper-based electronic devices. A number of solution printing techniques have therefore been developed for the fabrication of paper-based wearable devices as they are typically fast, inexpensive, and easily customizable. The existing techniques include inkjet printing (Choi et al., 2016; Huang et al., 2014; Lessing et al., 2014), screen printing (Adkins et al., 2017), spray coating (Asadpoordarvish et al., 2015), vacuum filtration (Li et al., 2014), pen writing (Liao et al., 2015, 2020; Russo et al., 2011; Xu et al., 2020b; Zheng et al., 2011), and dip coating (Ding et al., 2016; Gong et al., 2014). Also, *in situ* synthesis of functional materials by chemical reduction or polymerization is another alternative strategy (Zhang et al., 2018). Selection of printing techniques should be based on the demands for lateral resolution, printed thickness, homogeneity, printing speed, materials, and ink properties. Although optimization by mixing with mineral fillers has already been made in commercial copy paper to minimize surface roughness, challenges still remain when printing nanomaterial-based inks. For example, ink absorption into the porous substrate typically occurs as a result of the capillary force from micro-sized pores (Choi et al., 2016). Furthermore, nonuniform pore size distribution within the paper matrix causes random spreading of the ink droplets, together with its hygroscopic expansion, thereby leading to decreased lateral resolution (Choi et al., 2016; Lessing et al., 2014). Some methods have been proposed to alleviate this issue, such as surface silanization of paper with fluoroalkyl trichlorosilanes (Lessing et al., 2014) and surface coating with a primer layer where cellulose nanofibrils are deposited to form a well-developed nanoporous structure with minimized surface roughness (Choi et al., 2016).

**Figure 2:**
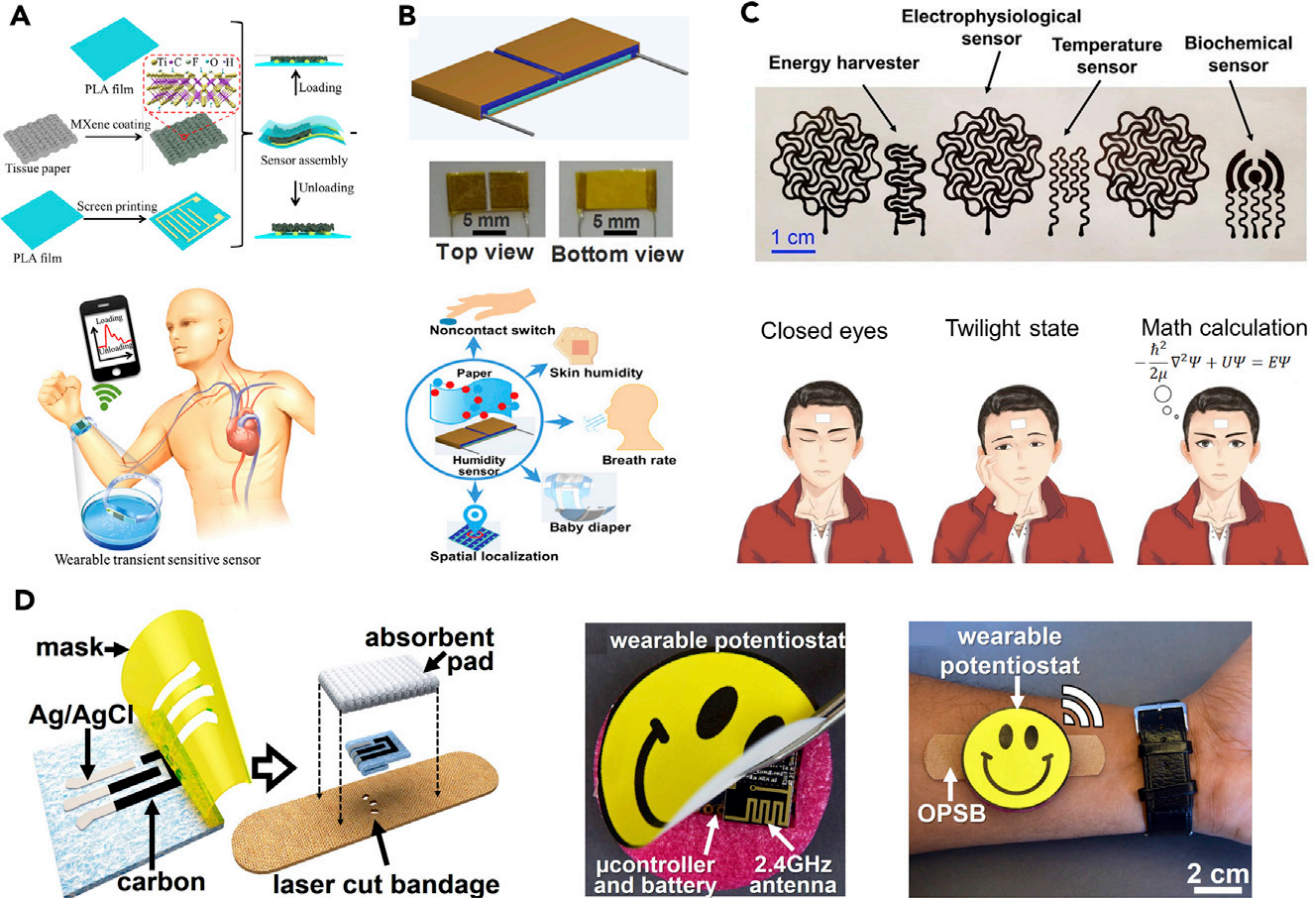
Paper-based wearable biosensors (A) Paper-based wearable pressure sensor for pulse waveform measurement. Adapted with permission from Guo et al. (2019). Copyright 2019, American Chemical Society. (B) Paper-based wearable humidity sensors for multiple biomedical applications. Adapted with permission from Duan et al. (2019). Copyright 2019, American Chemical Society. (C) Pencil-paper-enabled multimodal wearable device and its application in monitoring of the human mental state. Adapted with permission from Xu et al. (2020b). Copyright 2020, National Academy of Sciences. (D) Paper-based wearable smart bandage for electrochemical sensing. Adapted with permission from Pal et al. (2018). Copyright 2018, Elsevier.

During the past several years, the studies on paper-based wearable electronics have grown substantially, aiming to collect a variety of information such as temperature (Xu et al., 2020b), strain (mainly bending strain) (Hua et al., 2016; Liao et al., 2015), pressure (Gao et al., 2019a; Gong et al., 2014; Guo et al., 2019; Zhong et al., 2015), light (Lin et al., 2017; Pataniya and Sumesh, 2020), biopotential (Sadri et al., 2018; Xu et al., 2020b), pH (Xu et al., 2020b), gas (Huang et al., 2014; Lin et al., 2014; Liu et al., 2014; Mirica et al., 2012, 2013), humidity and respiration (Duan et al., 2019; Güder et al., 2016), and biochemical compositions (Gong and Sinton, 2017; Pal et al., 2018). In these devices, paper can serve as either breathable and biocompatible supporting substrates or active materials. Leveraging the piezoresistive characteristic of many functional materials, such as carbon-based materials, metals, metal oxides, and conductive polymers, numerous paper-based strain and pressure sensors have been developed (Mahadeva et al., 2015). The enabled devices can provide real-time and continuous monitoring of many vital signals, such as respiration, pulse waveform, and acoustic vibration (Figure 2A) (Güder et al., 2016; Tao et al., 2017). Leveraging its hygroscopic nature, cellulose paper has been used to make humidity sensors for various applications in noncontact switch, skin humidity, breath rate, and baby diaper (Figure 2B) (Duan et al., 2019). Moreover, paper-based VOCs (volatile organic compounds) sensors are emerging as an inexpensive and versatile alternative of conventional bulky and expensive gas chromatography mass spectroscopy (GC-MS) technique. The applications include medical diagnosis, environmental monitoring, and food quality assessment. For example, breath VOCs analysis has been employed for noninvasive diagnostic of a breadth of diseases including ovarian carcinoma, cancers, and end-stage renal disease (Broza et al., 2019). By modifying paper with various functional materials (e.g., carbon nanotubes, graphene, polypyrrole, and pencil leads), numerous paper-based chemiresistive VOCs sensors have been developed for analysis of ethanol, NO_2_, and NH_3_ (Mirica et al., 2012; Tai et al., 2020). The enabled paper-based VOCs sensors generally demonstrate good reproducibility and sensitivity (down to ∼80 ppb) (Liu et al., 2014), which largely depends on the active sensing materials.

In addition to piezoresistive and chemiresistive sensors, biopotential sensors that monitor the electrical properties of biological tissues provide a wealth of valuable information. For example, electrocardiogram (ECG) represents the electrical activity of heart muscle and remains an essential role in cardiac assessments. To achieve high-fidelity recording of electrophysiological signals, an intimate contact between the sensing electrodes and skin is required. Although paper is generally not sticky, silicone adhesive can be spray coated on its surface to resolve this issue (Xu et al., 2020b). The resulting paper substrate exhibited reliable and robust adhesion with skin while preserving its intrinsic breathability. In addition, there are various types of commercial adhesive cellulose papers (e.g., Aquasol adhesive water-soluble paper, Post-it Notes paper) that can meet this particular requirement without needing additional coating of silicone adhesive. Owing to low-cost resources, ease of fabrication, and abundant potential designs, paper-based wearable electronics show great potentials in electrical and optical stimulation, temperature, ECG, EMG (electromyogram), and EEG (electroencephalogram) recordings (Figure 2C) (Broza et al., 2019; Xu et al., 2020b).

Given the rich surface chemistry, intrinsic hydrophilicity, and hierarchically porous structure, cellulose paper is particularly attractive for biochemical sensors. The hydroxyl groups together with micro-sized pores provide the capillary force that drives the transportation of biofluids, which provides inherent capability for microfluidic devices. By selective surface functionalization with hydrophobic moieties, microfluidic channels can be constructed on paper (Mahadeva et al., 2015). Although continuous operation driven by the capillary force in a long-term manner has been rarely demonstrated by paper-based microfluidic devices, the physiologically relevant pressure from sweat glands (∼2 kPa) may enable continuous sweat transportation as demonstrated by elastomer-based microfluidic devices (Reeder et al., 2019; Yang et al., 2020; Yu et al., 2020). Although colorimetric detection is widely studied, electrochemical sensing is highly desirable owing to its quantitative analysis and insensitivity to light, dust, and insoluble compounds. This technique is extensively explored for a number of analytes such as glucose, cholesterol, drugs, pH, uric acid, potassium ferricyanide, L-lactate, and alcohol in blood, urine, and sweat samples (Nie et al., 2010; Pal et al., 2017, 2018; Xu et al., 2020b). For example, a paper-based wearable omniphobic smart bandage (OPSB) has been developed for the detection of pH, uric acid, and potassium ferricyanide in human open wounds, where carbon and silver/silver chloride inks are screen printed as working, counter, and reference electrodes, respectively (Figure 2D) (Pal et al., 2018). Subsequent interfacing the OPSB with a wearable potentiostat, battery, and antenna completed the device fabrication, enabling a wearable system for the early detection of pressure ulcers (Figure 2D). Paper-based point-of-care diagnostic devices are well studied in terms of the sample collection and analysis. Such biosensing applications can be exemplified by modifying paper electrodes with ion-selective membranes for potentiometric ion sensors. Various ion-selective electrodes are adopted for the detections of Cd^2+^, Ag^+^, K^+^, NH_4_^+^, Cl^−^, and Na^+^ (Hu et al., 2016; Mensah et al., 2014; Novell et al., 2012; Sakata et al., 2020). Given the intrinsic high porosity of cellulose paper substrate, these devices usually possess enhanced active surface area and therefore exhibit higher sensitivity. Of note, direct pencil drawing has also been demonstrated for the fabrication of wearable sweat sensors (Xu et al., 2020b). Compared with other often-used microfluidic devices (Bandodkar et al., 2019; Reeder et al., 2019; Yang et al., 2020), cellulose paper-based microfluidic devices exhibit distinct features, including (1) flexibility and breathability; (2) disposability; (3) inexpensive and scalable manufacturing process. However, challenges remain as encapsulation is required to minimize sample contamination and cross talk of different biosensors. Besides, the reproducibility and complicated calibration procedures are still main issues of paper-based biochemical sensors that need to be addressed by optimizations and innovations in the manufacturing process.

As compared with traditional wearable electronic devices, cellulose paper-based electronics typically exhibit comparable performances in terms of the sensitivity and signal quality (i.e., signal-to-noise ratio) since similar active materials are generally used in both paper-based and traditional wearable electronics. This can be exemplified by paper-based electrophysiological sensors, which exhibit negligible difference in signal quality with conventional gel electrodes (Xu et al., 2020b). In addition, surface modifications with hydrophobic moieties can substantially improve the stability of paper-based wearable devices. However, their long-term stability and robustness under wetting environments and repeated mechanical deformations are rarely studied, which are worth further investigations.

### Energy harvesters

**Figure 3:**
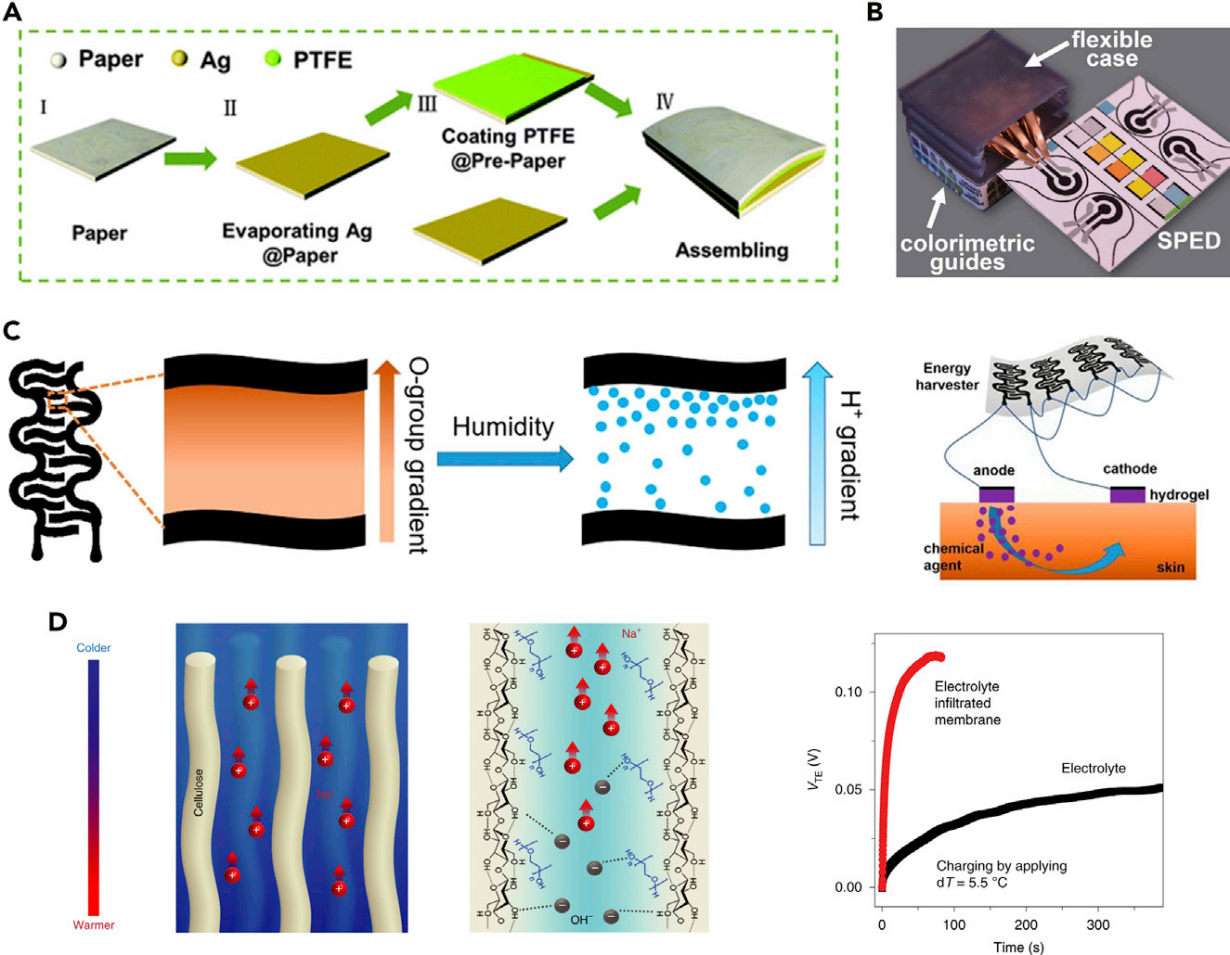
Paper-based energy harvesters (A) Schematic illustrations of the fabrication process of paper-based electrostatic nanogenerators. Adapted with permission from Zhong et al. (2013). Copyright 2013, Royal Society of Chemistry. (B) An electrochemical sensor powered by a paper-based triboelectric nanogenerator. Adapted with permission from Pal et al. (2017). Copyright 2017, Wiley-VCH Verlag GmbH & Co. KGaA, Weinheim. (C) Schematic illustrations of a paper-based humidity energy harvester for a self-powered transdermal drug-delivery system. Adapted with permission from Xu et al. (2020b). Copyright 2020, National Academy of Sciences. (D) Paper-based ionic conductors for thermoelectric heat harvesting. Adapted with permission from Li et al. (2019). Copyright 2019, Nature Publishing Group.

Wearable electronics require electric power to support sensing, data communication, and signal conditioning. Current incorporation of batteries and coin cells into skin-like wearable electronics pose challenges as a result of their mismatch in form factors (Bandodkar et al., 2020). A self-powered wearable system that harvests energy from body motion, sweat, ambient light, and moisture therefore emerges as an economically viable, sustainable solution (Bandodkar et al., 2020; Park et al., 2018; Song et al., 2020; Xu et al., 2020b; Yu et al., 2020). Given wide availability, disposability, and biocompatibility of cellulose paper, much progress has been achieved for paper-based energy-harvesting devices. These power generators generally rely on triboelectric, thermoelectric, hygroelectric, piezoelectric, and electrostatic effects. For example, cellulose paper easily donates electrons when rubbed with materials that are likely to gain electrons such as polyvinylidene fluoride (PVDF). Leveraging this unique property, a large variety of triboelectric nanogenerators has been developed (Feng et al., 2016; Guo et al., 2017; Xia et al., 2018; Zhang et al., 2017a, 2017b). In addition to cellulose paper supporting substrates, nanogenerators generally require additional modifications or active layers. For example, conductive electrodes and triboelectric pairs are required in electrostatic and triboelectric nanogenerators (Figure 3A) (Guo et al., 2017; Zhong et al., 2013). When gum wrappers are used, however, additional preparation of conductive electrodes is eliminated since aluminum foil coated on the backside can serve as conductive pathways (Feng et al., 2016). The resulting mechanical energy harvesters can generate instant and pulsed voltage output (hundreds of volts), which requires voltage rectification before practical uses. A systematic integration of paper-based triboelectric nanogenerators with paper-based microfluidic biochemical sensors provides a proof-of-concept self-powered wearable device for point-of-care applications (Pal et al., 2017).

The rich surface chemistry of cellulose paper has also been explored for moisture-induced energy generation. The abundant hydroxyl and carboxyl groups, which naturally exist on paper, provide exciting opportunities for ambient humidity-related applications. Water absorption by hydrophilic moieties induces the dissociation of surface functional groups, thereby releasing a large number of positive ions (i.e., protons) (Bai et al., 2019). If there is an inherent concentration gradient of oxygen-containing groups, chemical potential difference spontaneously forms and drives proton movement (Figure 3C). This charge carrier transportation leads to charge separation and therefore generates voltages and currents in an external circuit. Leveraging this unique feature, hygroelectric generators prepared by either generating asymmetric moisture levels or constructing a heterogeneous hydrophilic functional groups have been reported (Gao et al., 2019b; Xu et al., 2020b). Unlike mechanical energy harvesters, moisture-induced energy generators can produce continuous electrical output in the range of hundreds of millivolts that are suitable for direct use or storage. A proof-of-concept self-powered iontophoretic transdermal drug-delivery system was demonstrated using the moisture-induced electric energy (Figure 3C) (Xu et al., 2020b).

Besides, thermoelectric generators (TEGs) that can convert low-grade heat into electric energy is of considerable interest for wearable electronics. Like other energy generators, paper-based TEGs can be fabricated by integrating with conventional p- and n-type inorganic thermoelectric composites into a porous structure (Rojas et al., 2017; Zhao et al., 2019). These TEGs generally exhibit output voltages in the range of tens of millivolts and output power of tens of nanowatts, which are, however, still far from practical wearable applications. Introduction of electrolyte into a cellulosic membrane with oxidized and aligned cellulose molecular chains results in sub-nanoscale confinement of ions and therefore induces selective ion diffusions that essentially enhance thermoelectric performance (Figure 3D) (Li et al., 2019). Yet, this process requires a particular cellulose (i.e., type II cellulose) and does not occur in natural wood or type I cellulose.

### Energy storage devices

Despite recent progresses, challenges still remain as current paper-based energy generators have numerous limitations such as inadequate power output, low energy efficiency, and intermittent energy generation. A wearable energy storage system is therefore required to provide stable and continuous power supply that can meet the demand for sensing, data communication, and signal conditioning. Various energy storage platforms have been developed to meet this requirement, among which batteries and supercapacitors stand out. Cellulose paper is promising to make lightweight, low-cost, and disposable self-powered wearable electronics. Moreover, hierarchically porous cellulose fibrous networks and hygroscopic features contribute to the penetration and absorption of conductive fillers, thereby enhancing their interface interactions and facilitating electrons/ions transfer, which is desirable for high-performance wearable batteries.

**Figure 4:**
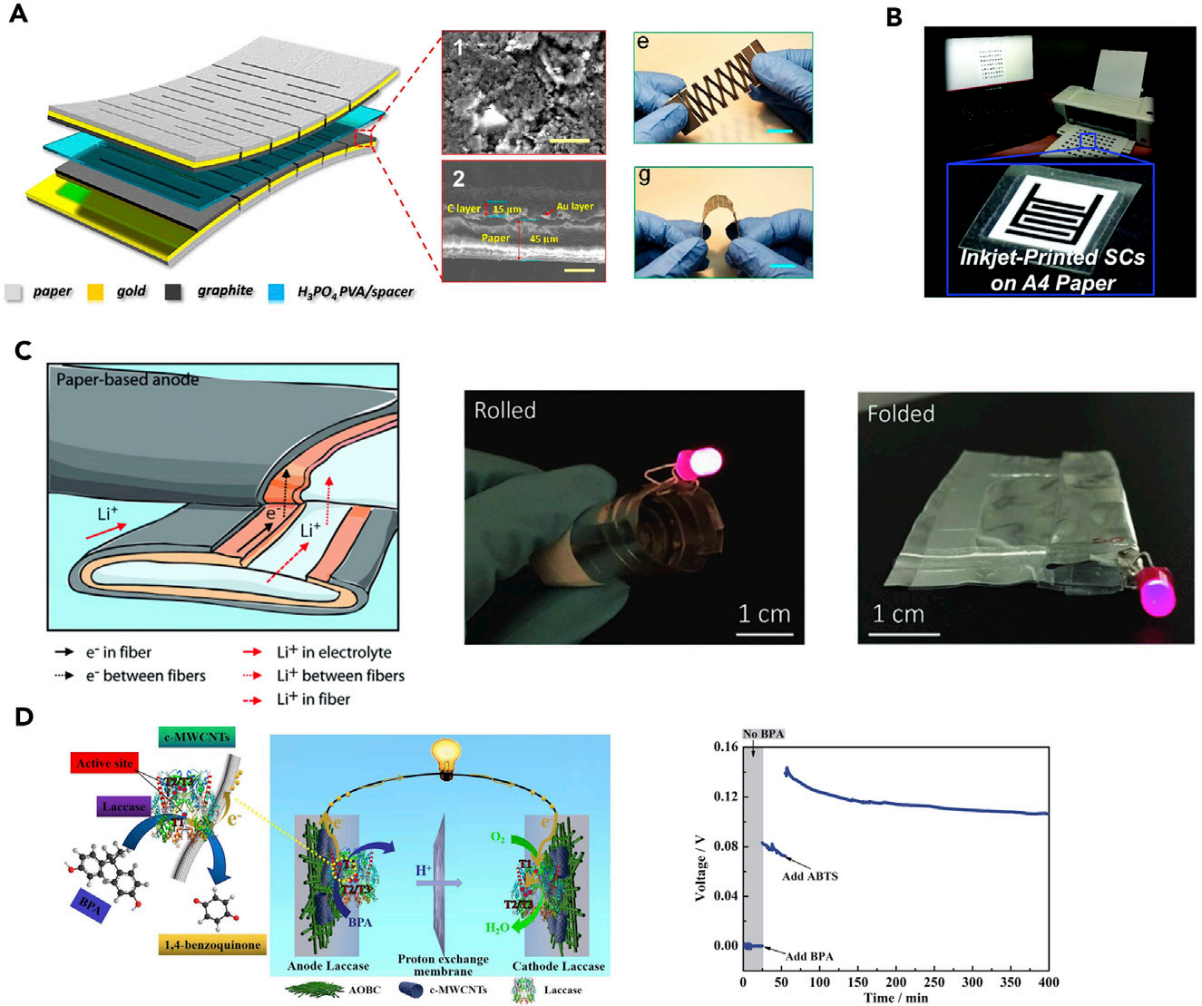
Paper-based energy storage devices (A) Paper-based supercapacitors with kirigami designs. Adapted with permission from Guo et al. (2016). Copyright 2016, American Chemical Society. (B) A solid-state supercapacitor inkjet printed on paper. Adapted with permission from Choi et al. (2016). Copyright 2016, Royal Society of Chemistry. (C) Copper-plated paper for foldable lithium ion batteries. Adapted with permission from Wang et al. (2018). Copyright 2018, Wiley-VCH Verlag GmbH & Co. KGaA, Weinheim. (D) Schematic illustrations and performance characterizations of paper-based enzymatic cellulose biofuel cell with bisphenol A as fuel. Adapted with permission from Li et al. (2020). Copyright 2020, Elsevier.

Cellulose paper-based supercapacitors, Li-ion batteries, Li-S batteries, Li-O_2_ batteries, and biofuel cells have been extensively studied (Yao et al., 2017; Zhang et al., 2015, 2018). For example, paper-based supercapacitors have been fabricated by a facile graphite pencil drawing (Guo et al., 2016; Yao et al., 2013; Zheng et al., 2011), vacuum filtration (Li et al., 2014), or a scalable inkjet printing (Choi et al., 2016), where conductive electrodes (carbon nanotubes, graphene, silver nanowires, polypyrroles, and polyanilines) are typically employed (Zhang et al., 2015) (Figures 4A and 4B). Considerable research efforts have been made due to their outstanding reversibility, high-power density, long-life cycles, and safe operation. Of note, the kirigami and origami designs can expand the stretchability of paper-based wearable devices (Figure 4A). Li-ion batteries are another powerful and versatile platform that has already shown promise in portable electronic devices owing to their outstanding energy density, high operation voltage, low self-discharging rate, lack of memory effect, long life cycles, and environmentally friendly features (Zhao et al., 2020). Li-ion batteries generally comprise a LiCoO_2_ cathode, a graphite anode, and a separator saturated with a liquid organic electrolyte. An example using copper nanoparticles-plated cellulose paper as the anode is provided in Figure 4C, which illustrates the mechanical flexibility of the resulting battery (Wang et al., 2018). Proper packaging and encapsulations are also essential to construct an integrated wearable self-powered system. To achieve a sustainable and clean energy supply, biofuel cells that leverage the oxidation and reduction processes of renewable fuels can generate energy from biological matters (Song et al., 2015). Representative examples include alcohols, sugar, and amine substances, such as glucose, ethanol, pyruvate, and lactate (Escalona-Villalpando et al., 2019; Li et al., 2020), which can potentially enable future sweat-powered skin-interfaced electronics or blood-powered implantable devices (Bandodkar et al., 2017, 2020). Integrating biofuel cells with cellulose paper substrates, although still in their infancy, can provide a promising alternative for the fabrication of completely sustainable and disposable self-powered wearable electronics. An enzymatic biofuel cell that employs laccase as biocatalyst can generate power (power density: 1.897 W m^−3^; open circuit voltage: 0.14 V) from waste water (Figure 4D) (Li et al., 2020). The highest output power density (∼200 μW cm^−2^) is achieved on a cellulose filter paper using glucose as the biofuel (Desmet et al., 2016; Wang et al., 2014).

### Other applications

**Figure 5:**
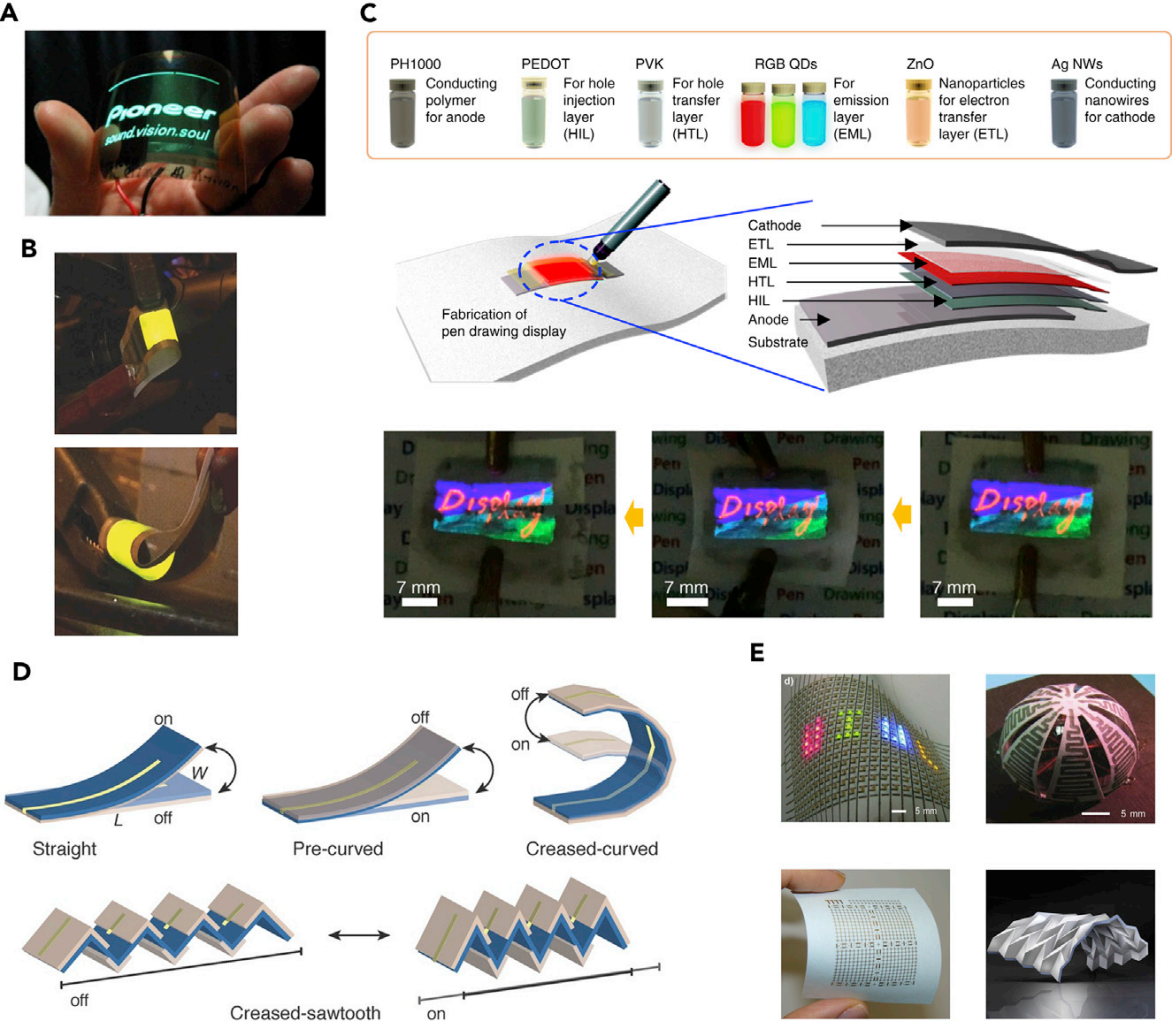
Other applications of paper-based electronics (A) An optical image of an organic light-emitting diode on transparent, resin-infiltrated cellulose paper. Adapted with permission from Okahisa et al. (2009). Copyright 2009, Elsevier. (B) Photographs of surface-emitting light-emitting electrochemical cells on flexible cellulose paper substrates. Adapted with permission from Asadpoordarvish et al. (2015). Copyright 2015, Wiley-VCH Verlag GmbH & Co. KGaA, Weinheim. (C) Schematic illustrations and photographs of the pen-drawing display on paper. Adapted with permission from Jeong et al. (2019). Copyright 2019, Nature Publishing Group. (D) Schematic illustrations of paper-based hygroexpansive electrothermal actuators. Adapted with permission from Hamedi et al. (2016). Copyright 2016, Wiley-VCH Verlag GmbH & Co. KGaA, Weinheim. (E) Optical images of paper-based flexible electric circuits, antenna, transistors, and photodetectors. Adapted with permission from Lin et al. (2017); Russo et al. (2011); Zschieschang and Klauk (2019). Copyright 2017, American Chemical Society; copyright 2011, Wiley-VCH Verlag GmbH & Co. KGaA, Weinheim; copyright 2019, Royal Society of Chemistry.

Display is one basic building block of next-generation wearable electronics. Also, wearable display can bridge human-device interactions. Transforming conventional rigid displays into flexible and wearable forms provides fascinating opportunities for wearable technologies (Shi et al., 2021). Regular paper is inherently not suitable for organic light-emitting displays owing to its surface roughness and opaque feature. Highly transparent cellulose nanopaper with low haze and smooth surface has been explored for flexible displays (Figure 5A) (Okahisa et al., 2009). The nanopaper comprises nanofibrillated celluloses that are assembled into densely packed thin films, thereby substantially improving optical transmittance and reducing surface roughness (Fang et al., 2014; Zhu et al., 2016). The dimensional reduction of microscale fibers in traditional copy paper also is effective for minimizing surface roughness. Besides, filling hierarchically porous cavities with polymers provides an alternative approach to reduce surface roughness for optoelectronic applications (Ha et al., 2018). Light-emitting electrochemical cells can overcome these issues as a result of *in situ* formation of a light-emitting p–n junction (Figure 5B) (Asadpoordarvish et al., 2015). Commonly used cellulose paper with microscale fibers serving as a supporting substrate for flexible organic light-emitting displays has also been reported (Jeong et al., 2019). Specifically, a facile pen-drawing approach is employed. Here, conducting and light-emitting functional materials are loaded into various drawing pens for anode, hole injection layer, hole transfer layer, emission layer, electron transfer layer, electron injection layer, and cathode, respectively (Figure 5C). Despite these progresses, paper-based flexible displays still face challenges in terms of thermal stability and mechanical durability.

Given the hygroscopic nature from surface hydroxyl groups, cellulose paper is also exploited for soft actuators. Upon moisture exposure, the cellulose matrix expands or contracts in response to humidity variations (Amjadi and Sitti, 2016). The moisture content in the paper can be modulated by electrothermal heating, which results in dimensional deformations when a strain-constraining layer is integrated (Figure 5D) (Hamedi et al., 2016). Cellulose paper-based soft actuators can potentially offer exciting opportunities for liquid transport control in microfluidic devices in terms of micromachine assembly and microactuation (Hamedi et al., 2016). Actuation mechanisms that utilize magnetic and electrostatic fields were also demonstrated. However, these methods usually suffer from the requirement of magnetic additives, high voltage supply, and insufficient movement.

Also, paper's appealing properties make it suitable for a number of other applications. These can be exemplified by transistors (Zschieschang and Klauk, 2019), electric circuits (Hyun et al., 2013; Russo et al., 2011), radio frequency identification (RFID) tags (Wang et al., 2019), antennas (Inui et al., 2015; Xu et al., 2020b), photodetectors (Gomathi et al., 2017; Lin et al., 2017; Pataniya and Sumesh, 2020), and artificial nerve (Liao et al., 2020) (Figure 5E).

### Toward highly integrated wearable systems

**Figure 6:**
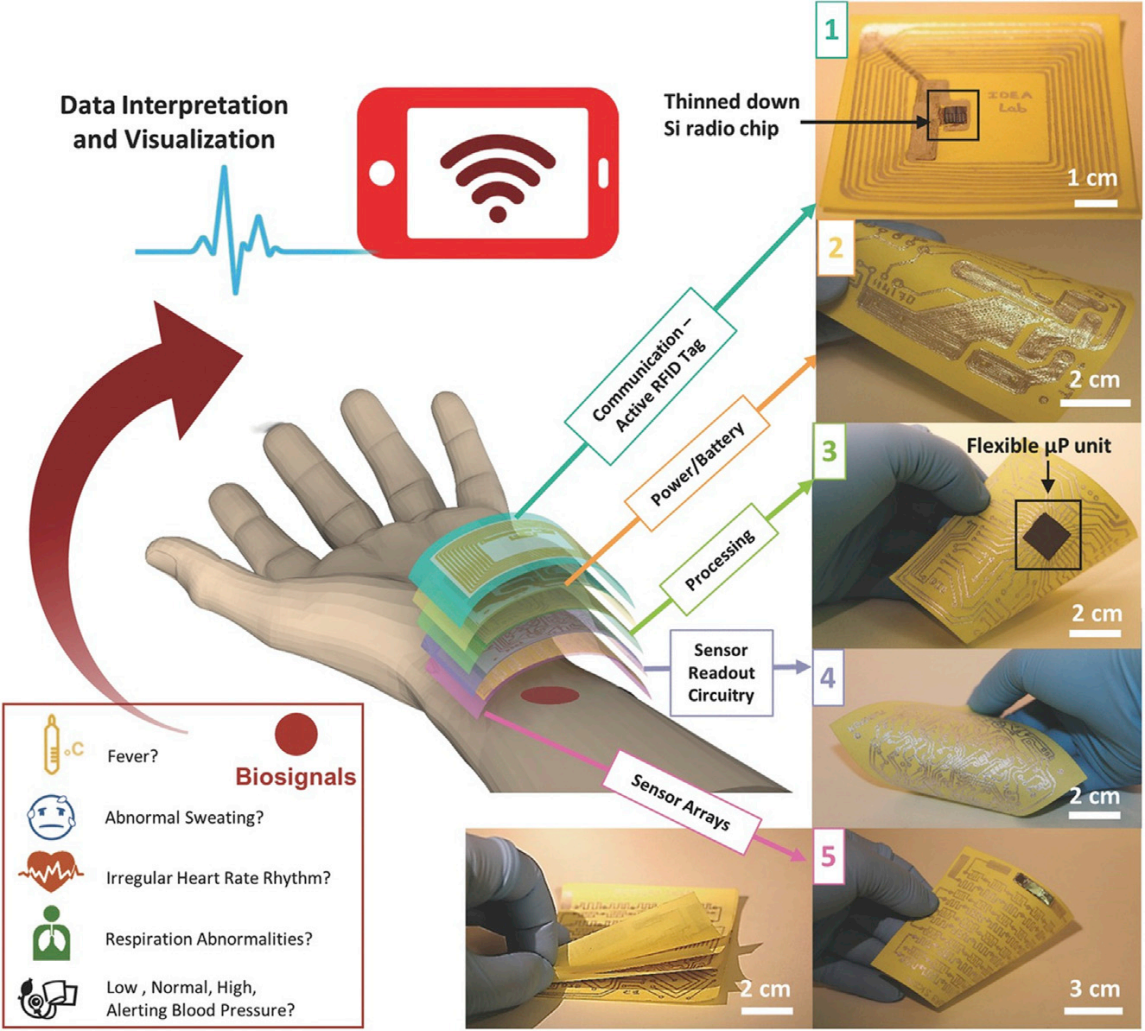
A paper-based highly integrated wearable healthcare monitoring system Adapted with permission from Nassar et al. (2017). Copyright 2017, Wiley-VCH Verlag GmbH & Co. KGaA, Weinheim.

During the past several years, we have witnessed the significant advancements of paper-based electronics in a variety of research realms, including sensing, energy generation and storage, optoelectronics, and data transmission. In the future, a paper-based integrated wearable electronic system is highly desirable for skin-interfaced biomedical applications. Ideally, the system should comprise biodegradable cellulose supporting substrates and minimal amount of nondegradable functional materials to minimize environmental impact. An all-paper supported wearable system, consisting of vital signal sensing, data processing, power supply, and signal transmission, was recently demonstrated (Figure 6) (Nassar et al., 2017). This highly integrated system uses Post-it Note paper as the supporting substrate, integrated with RFID tag for radio communication, power management circuitry, silicon-based microprocessor (μP), and various sensors. This paper-based wearable system is capable of monitoring a broad spectrum of vital biosignals (e.g., temperature, respiration, heart rates, blood pressure, and sweating) and can be interfaced with a smartphone for wireless data collection, interpretation, and visualization.

## Challenges and conclusion

Given the promising properties of cellulose paper in terms of inexpensive and scalable manufacturing, biodegradability, disposability, biocompatibility, breathability, and rich surface chemistry, a variety of paper-based devices have been developed, ranging from sensing, energy storage and generation, optoelectronics, and soft actuators to wireless data communication. These research advancements are driven by the attempt to seek for a more sustainable, disposable, and environmentally friendly alternative of currently polymer-based flexible electronics. Although most current studies use pristine cellulose paper as the supporting substrate, its abundant surface hydroxyl groups and intra- and inter-molecular hydrogen bonds offer many exciting opportunities for hybridization with other functional moieties to meet requirements of customized applications. These modifications generally contribute to the enhanced mechanical strength, decreased surface roughness, and improved thermal and chemical stability against inorganic and organic solvents.

Although a broad range of proof-of-concept paper-based wearable devices have been demonstrated, many challenges still remain, especially for the transition from laboratory-based demonstrations to practical uses. These obstacles involve material and chemistry aspects, which are critical for cellulose paper as a sustainable alternative of polymer substrates. For example, although the hierarchically porous structure formed by the entangled fibrous cellulose network offers excellent breathability for skin-interfaced electronics and large surface area for energy storage-related applications, the porosity inevitably results in the incompatibility with some solution-printing techniques (e.g., inkjet printing) owing to uncontrolled ink spreading. This will further cause conductivity deterioration as a result of ink penetration into a deeper depth of the paper. Moreover, as compared with conventional miniaturized electronic devices, the printing resolution on cellulose paper has yet met the demand for future highly integrated electronics. In addition, the microscale surface roughness and intrinsic opaque nature of commercial cellulose paper limit its application in optoelectronics that typically requires high optical transmittance and low surface roughness. Unlike conventional polymers, pristine cellulose paper is not mechanically or thermally stable. Dimensional deformations caused by moisture absorption will compromise device performance and the reliability of the recorded data.

Although there are multiple options available in the market, adhesiveness is of superior importance for skin-interfaced wearable electronics since conformal contact with human skin is a critical factor of high-fidelity biosignal recording. Motion-induced artifacts during regular human activities will cause increased noise level and signal degradation. Examples of adhesive papers used for wearable electronics include Post-it Note paper and water-soluble adhesive paper. The stickiness is often associated with a thin layer of adhesive polymer, which comes at the sacrifice of breathability to some extent. This issue can probably be addressed by innovative material design that can provide the required adhesiveness while simultaneously preserving the porous structure. While adhesive paper is commercially available, the deposition of conductive electrodes that are typically non-sticky will compromise the overall interface adhesion. Adopting an open-mesh serpentine layout is an effective strategy since this can maximize the proportion of nonconductive adhesive area and minimize deformation-induced local strains. Alternatively, it is desirable to develop adhesive conductive composites, comprising conductive fillers and adhesive polymers, as sensing electrodes.

In the light of an eco-friendly society, paper-based wearable electronics will be completely disposable when biodegradable electronic materials are used for device fabrication. Although various emerging carbonaceous nanomaterials bring promising routes toward paper-based all-biodegradable device, the performance is yet comparable with conventional nonbiodegradable materials. Using some nonbiodegradable components is still indispensable for high-performance tasks, such as data processing and management. Besides, the research of paper-based, highly integrated, wearable electronic system is still in the infant stage, which requires more exploration of fundamental science and technologies at the molecular level and all-paper based system assembly.

These obstacles inevitably impede the advancement and commercialization of cellulose paper-based wearable electronics. Nevertheless, they can be addressed through judicious material innovation, structural engineering, and advancement of other related realms (e.g., fabrication techniques) to satisfy specific demands for various arising applications. For example, stability issues in wet environments can be alleviated through proper chemical functionalization (e.g., silanization); substantial surface roughness and low transmittance can be resolved by downscaling constituent fibrous diameters; inadequate adhesion to skin can be enhanced by hybridizing cellulose paper with adhesive chemicals or proper bioinspired structure engineering (e.g., bioinspired dry adhesive structure [Chun et al., 2018; Wang, 2018]); low stretchability can be improved by introducing kirigami design. In addition to conventional copy paper, an expanded array of nanopapers comprising nanofibrillated cellulose is emerging thanks to extensive research efforts on fundamental research studies in terms of materials and chemistry innovations. These nanopapers have raised substantial interests as they bypass existing issues of microscale cellulose counterparts, such as considerably improved surface roughness and transparency. However, microscale and nanoscale cellulose papers are only negligibly stretchable, which might limit their wide adoption in wearable electronics. Nevertheless, we anticipate fertile opportunities of cellulose papers in sustainable wearable electronics considering their mechanical, economic, and environmental benefits.
